# Effiziente Triage und verbesserte Patientenversorgung: Fünf Jahre Teledermatologie in der Steiermark: Efficient triage and improved patient care: Five years of teledermatology in Styria

**DOI:** 10.1111/ddg.70097x

**Published:** 2026-11-10

**Authors:** Arzberger Edith, Bordag Natalie, Hofmann‐Wellenhof Elena, Freytag Leo, Hofmann‐Wellenhof Rainer

**Affiliations:** ^1^ Univ.‐Klinik für Dermatologie und Venerologie Medizinische Universität Graz Graz Österreich; ^2^ e‐derm‐consult Graz Österreich

**Keywords:** Asynchrone Telemedizin, Dermatoskopie, Kooperation zwischen Primärversorgung und Dermatologie, ländliche Gesundheitsversorgung, Patientenzufriedenheit, Teledermatologie, Triage, Asynchronous telemedicine, Dermoscopy, Patient satisfaction, Primary caredermatology collaboration, Rural health services, Teledermatology, Triage

## Abstract

**Hintergrund:**

Zur Verbesserung der dermatologischen Patientenversorgung in ländlichen Gebieten wurde am 1. Januar 2020 das Projekt „Teledermatologie in der Steiermark“ gestartet. Allgemeinmediziner (AM) wurden digital mit Dermatologen (DERM) vernetzt. Das Projekt wurde vom Gesundheitsfonds Steiermark, der steirischen Ärztekammer, der Österreichischen Gesundheitskasse Steiermark, der Universitätsklinik für Dermatologie Graz und der e‐derm‐consult GmbH unterstützt.

**Patienten und Methodik:**

AM übermittelten klinische Angaben und dermatoskopische Fotos ihrer Patienten mittels *store‐and‐forward* an DERM, die eine Diagnose und Therapieempfehlung erstellten. Die deskriptive statistische Analyse umfasste Fälle aus 5 Jahren ohne Referenzregion und bewertete die Akzeptanz bei Patienten und Ärzten anhand von Fragebögen.

**Ergebnisse:**

Bei 25% der 5119 Fälle bestätigten DERM die Diagnose der AM, während in 51% DERM eine andere Diagnose stellten und in 23% AM keine Diagnose angaben. Während in 19% keine Therapie benötigt wurde, wurden die meisten (61%) von AM behandelt nach teledermatologischer Konsultation. Überweisungen wurden bei 12% für einen dermatologischen Normal‐, 3% einen Akut‐ und 2% einen Kliniktermin benötigt. Es wurden Fälle aus dem gesamten dermatovenerologischen Spektrum behandelt, wovon rund 34% der Fälle Neoplasien waren. In den 692 retournierten Fragebögen lag die Patientenzufriedenheit bei über 95%.

**Schlussfolgerungen:**

Rund 17% der Patienten benötigten eine zusätzliche hautfachärztliche Untersuchung. Die um Monate schneller erhaltene Diagnose und die hohe Patientenzufriedenheit zeigen deutlich die Vorteile einer digitalen dermatologischen Konsultation.

## Summary

**Background:**

To improve dermatological care in underserved rural regions, the “Teledermatology in Styria” project launched on January 1^st^, 2020. General practitioners (GP) were digitally connected with dermatologists (DERM), supported by Styrian Health Fund, Styrian Medical Association, Austrian Health Insurance Fund Styria, Universities’ Department of Dermatology, and e‐derm‐consult GesmbH.

**Patients and Methods:**

GPs submit clinical information and images (clinical, dermoscopic) of patient's skin conditions, via a “store‐and‐forward” service to DERM, who responded with diagnosis and treatment recommendations. The descriptive statistical analysis covered cases from five years without reference region and assessed patient and doctor acceptance through questionnaires.

**Results:**

From 5,119 cases DERM changed GPs diagnosis in 51%, no diagnosis was provided by GPs in 23% and only in 25% DERM confirmed GPs diagnosis. While 19% of all cases required no therapy, most cases (61%) were managed by their GPs following teledermatological consultation. Referrals included 12% routine dermatological appointments, 3% urgent appointments, and 2% hospital visits. The case spectrum covered all dermato‐venereological conditions, with only 34% neoplasms. Patient satisfaction exceeded 95%, based on 692 returned questionnaires.

**Conclusions:**

Only 17% of patients required additional dermatological examination after teledermatology. The months faster diagnosis and the high level of patient satisfaction highlight the benefits of teledermatological consultations.

## EINLEITUNG

Trotz guter medizinischer Versorgung in Österreich sind in der ländlichen dermatologischen Versorgung monatelange Wartezeiten die Regel. In der österreichischen Ausbildungsordnung für Allgemeinmedizin ist Dermatologie und Venerologie nur noch ein Wahlfach.^1^ Allerdings suchen 10 %–20 % aller Patienten von Allgemeinmedizinern (AM) die Ordination aufgrund eines dermatologischen Problems auf.^2^, ^3^, ^4^ Am häufigsten handelt es sich dabei um entzündliche Erkrankungen, wobei Hauttumoren auf dem Vormarsch sind,^5^, ^6^ bedingt durch Veränderungen im Lebensstil und in der Demographie.^6^, ^7^ Hautkrebs ist dank der Auflichtmikroskopie (Dermatoskopie) rasch und in frühen Stadien zu erkennen.^8^, ^9^ Dabei verkürzt die rechtzeitige Entfernung die Krankenstandsdauer, vermeidet teure Therapien in späteren Stadien und verbessert erheblich die Lebensqualität der Betroffenen.^10^, ^11^

Die Teledermatologie entwickelt sich vielerorts als ein unverzichtbarer und im Gesundheitswesen voll integrierter Bestandteil dermatologischer Versorgung,^12^, ^13^ besonders weil die Haut gut zugänglich und die Dermatologie ein morphologisches Fach ist. Praxis und Literatur belegen, dass die teledermatologische Diagnosestellung jener im Rahmen eines persönlichen Patient‐Arzt‐Kontaktes nicht unterlegen ist.^14^, ^15^, ^16^

Teledermatologie verbindet Dermatologen (DERM) digital mit Allgemeinmedizinern oder Patienten *(1)* asynchron in einer *Store‐and‐Forward* (SF)‐Technologie, *(2)* synchron in Echtzeit (*Realtime*)‐Applikationen (RA) oder *(3)* in Hybridmodellen.^13^, ^17^, ^18^ Alle Optionen haben spezifische Vor‐ und Nachteile. Die *Store‐and‐Forward*‐Technologie ist ressourceneffizient, technisch einfach und zeitunabhängig, während RA‐Anamnese in Echtzeit mit direkter Befundbesprechung ermöglicht. Eine Verbindung zwischen Dermatologen und Allgemeinmedizinern gewährleistet die medizinisch professionelle und schnelle Fallerfassung, während die Dermatologen‐Patienten‐Verbindung die Allgemeinmediziner entlastet.

In einer 2018 durchgeführten Umfrage zum Thema Teledermatologie nahmen 243 österreichische Dermatologen teil (47% m, 55% w). Rund die Hälfte gab an bereits Erfahrung auf dem Gebiet gemacht zu haben, wobei die Hälfte eher vom Einholen einer Zweitmeinung sprach, nur ein Drittel von einer Erstvisite. Für 73% wäre eine durch Teledermatologie herbeigeführte Arbeitsentlastung in der Routine vorstellbar.^19^

Ziel des Projekts „Teledermatologie in der Steiermark“ war es, die dermatologische Patientenversorgung in ländlichen Gebieten zu verbessern, aufbauend auf Erfahrungen aus Deutschland.^20^ Das Projekt startete in Liezen, einem dünn besiedelten, abgelegenen Bezirk, und wurde später auf Leibnitz, einen Bezirk mit ähnlicher Bevölkerungszahl in der Nähe von Graz, ausgeweitet.

## METHODIK

### Projektverlauf

Das Projekt wurde nach Prüfung durch die Ethikkommission der Medizinischen Universität Graz (EK 32‐228 ex 19/29) am 1. Januar 2020 gestartet. Der Gesundheitsfonds Steiermark hat das Projekt finanziell unterstützt, die Ärztekammer der Steiermark war Projektwerber, die Österreichische Gesundheitskasse erstattete die ärztliche Leistung, die Medizinische Universität Graz begleitete wissenschaftlich und e‐derm‐consult übernahm die technische Abwicklung.

Die 15–30‐minütige Einschulung der Allgemeinmediziner erfolgte meist persönlich und beinhaltete korrekte Bild‐ und Anfrageerstellung im Telederm‐System (Sicherheit siehe Online‐Supplement S1). Dies ermöglichte teledermatologische Anfragen als Alternative zur herkömmlichen Überweisung. Alle Allgemeinmediziner erhielten ein Tablet (iPad mini 5, Apple Inc., Cupertino, CA, USA) und ein mobiles, wartungsfreies Dermatoskop mit Polarisation (DermLite DL1, 3Gen, San Juan Capistrano, CA, USA) zur Bildaufnahme und Übermittlung an die Dermatologen (Online‐Abbildung S1). Nach der Fallübermittlung übermittelten Dermatologen eine Diagnose und Therapieempfehlung an die Allgemeinmediziner; Rückfragen waren möglich. Der Funktionsumfang der Software wurde kontinuierlich erweitert, wie zum Beispiel um Diktier‐ und erweiterte Suchfunktionen.

### Studienaufbau und Ablauf der Televisiten

Patienten mit unklaren Hautveränderungen wurden von den Allgemeinmedizinern über die Möglichkeit der Televisite aufgeklärt. Nach der schriftlichen Einwilligung erfassten die Allgemeinmediziner den Fall, inklusive Anamnese, Medikation sowie klinischer und dermatoskopischer Bilder (Abbildung 1). Die Allgemeinmediziner bewerteten ihren Zeitaufwand und die Benutzerfreundlichkeit von 1 (sehr gut) bis 5 (ungenügend). Die Dermatologen wurden per SMS oder E‐Mail informiert und lieferten Freitext‐Diagnosen und Therapieempfehlungen, bevorzugt innerhalb von zwei Werktagen. Die weitere Vorgehensweise wurde in sechs Triage‐Kategorien eingeteilt (Abbildung 3): *(1)* keine Therapie indiziert, *(2)* Therapie beim Allgemeinmediziner, *(3)* Normaltermin beim Dermatologen in <3 Monaten, *(4)* Akuttermin beim Dermatologen in <3 Tagen, *(5)* Zuweisung an die Klinik oder *(6)* Rückfragen/nicht erfasst. Mit *(6)* wurden zusätzliche oder bessere Bilder vom Dermatologen nachgefordert oder Befundbesprechung angefragt. Bei *(3–5)* wurden Terminvorschläge vom Dermatologen üblicherweise direkt mitgesendet. Bei *(2)* enthielt der Therapievorschlag im Freitext Angaben zur Dosierung und Dauer. Zum Fallabschluss bewerteten die Dermatologen ihren Zeitaufwand sowie Bild‐ und Anfragequalität vom Allgemeinmediziner von 1 (sehr gut) bis 5 (ungenügend), während die Allgemeinmediziner die Qualität der Antwort der Dermatologen bewerteten.

**ABBILDUNG 1:**
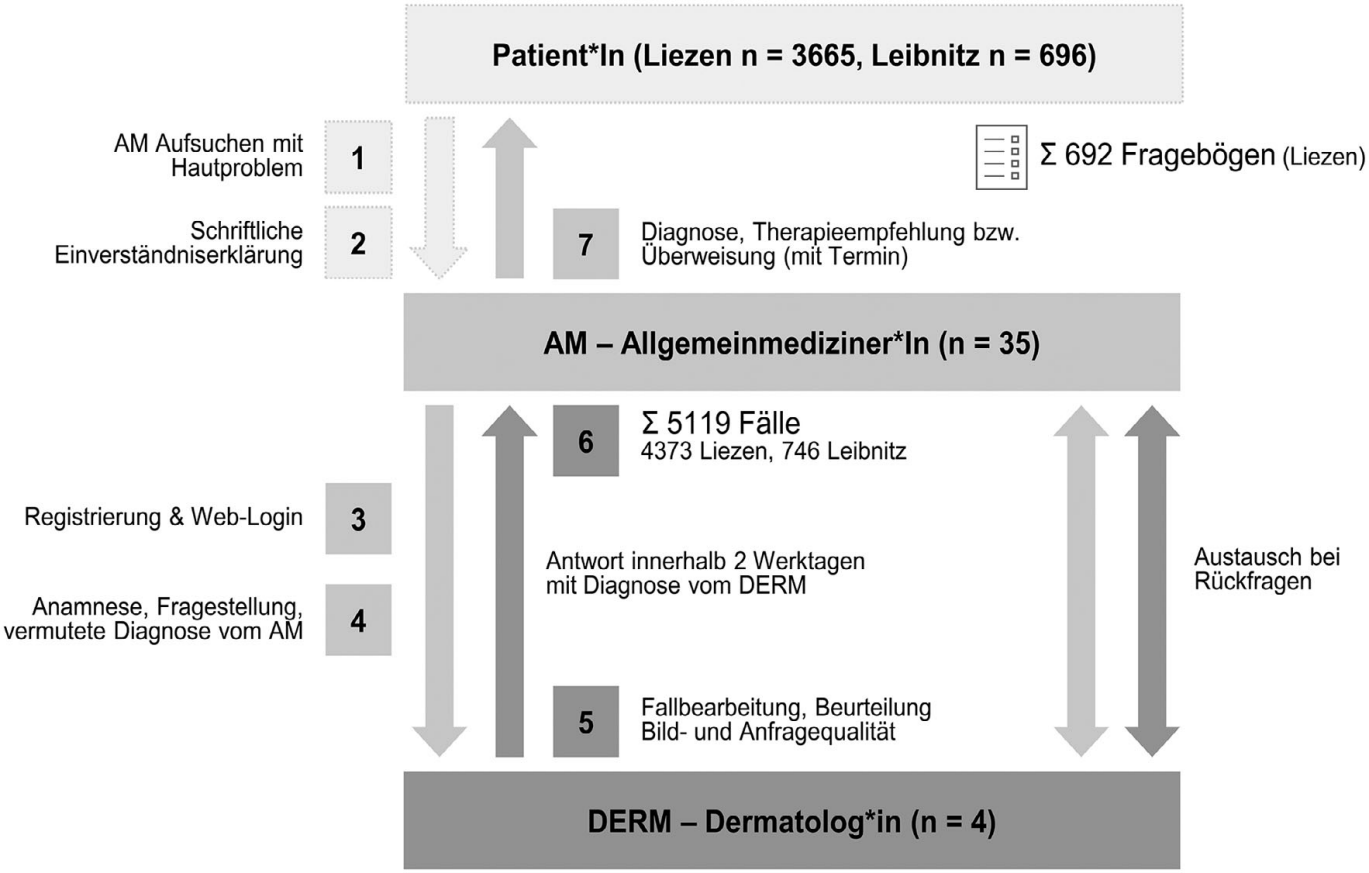
Schematische Übersicht über den Ablauf einer teledermatologischen Anfrage mit Anzahl der retournierten Fragebögen, Patienten und Ärzte.

Für die statistische Auswertung wurden die Diagnosen, in Anlehnung an ein Lehrbuch für Dermatologie und Venerologie,^21^ in fünf Gruppen unterteilt: entzündliche Hauterkrankungen (Infektionen, äußere und unklare Ursachen); Neoplasien der Haut (gut‐ oder bösartig); Gewebs‐ und regionsspezifische Hauterkrankungen; Erkrankungen des Gefäßsystems; Sonstige.

In Liezen wurde die Meinung der Patienten nach abgeschlossener Behandlung mittels Fragebogen erhoben. Allgemeinmediziner aus beiden Bezirken erhielten Fragebögen, dabei wurde unterschieden zwischen am Teleprojekt nicht oder bereits teilnehmenden Allgemeinmedizinern (Fragebögen siehe Online‐Supplement S1, Daten siehe Online‐Supplement S2).

### Projektkosten

Die Kosten für EDV‐Infrastruktur, Software, Hardware, Projektkoordination, Einschulung und laufende Betreuung der Mitwirkenden sowie der wissenschaftlichen Begleitung, Auswertung der Daten und Berichtslegung wurden vom Gesundheitsfonds des Landes Steiermark getragen. Die ärztliche Leistung der Behandlung der 5119 Fälle wurde von den Krankenkassen des Landes übernommen. Bei Mehrfachzuweisungen konnte pro Patient einmal im Quartal verrechnet werden, unabhängig vom Mehraufwand für Rückfragen pro Fall.

### Statistik

Für die statistische Analyse wurden alle Fälle im Zeitraum von 01.01.2020 bis 31.12.2024 exportiert. Technische Tests (41), Fehlanforderungen (7), Anforderungen mit fehlenden (8) oder unzureichenden Bildern (3) wurden ausgeschlossen, so dass 5119 Fälle von 4361 Patienten für die Analyse verwendet wurden. Zum Vergleich wurde die Altersverteilung der Gesamtbevölkerung von 2022 von Statistik Austria,^22^ herangezogen.

Die statistische Analyse erfolgte mittels R (v 4.4.0),^23^ und TIBCO Spotfire (v 14.4.0, TIBCO, Palo Alto, CA). Die Altersverteilung wurde mittels Pearson‐Chi‐Quadrat‐Test (stats::chisq.test()) untersucht, während die anschließenden post hoc paarweisen Vergleiche mittels epitools::oddsratio() und multipler Testkorrektur nach Benjamini‐Hochberg (BH) (stats::p.adjust()) analysiert wurden. Alle erhobenen Daten sind im Online‐Supplement S2 angefügt mit zufälligen, pseudonymisierten IDs, die niemandem außerhalb des Forschungsteams bekannt sind.

## ERGEBNISSE

### Projektverlauf

Das Projekt „Teledermatologie in der Steiermark“ startete am 1. Januar 2020 im Bezirk Liezen mit 15 Allgemeinmedizinern und zwei Dermatologen (Abbildung 1, Abbildung S1 im Online‐Supplement). Das Projekt wurde später auf insgesamt 35 Allgemeinmediziner und vier Dermatologen in Liezen und Leibnitz erweitert (Abbildung 2) und verzeichnete 5119 abgeschlossene Fälle innerhalb von 5 Jahren. Von den 35 Allgemeinmedizinern traten zwei in den Ruhestand, einer wurde Internist und sechs lehnten die Fortsetzung aufgrund des zusätzlichen Arbeitsaufwandes ab. Die frei gewordenen Plätze wurden entsprechend weiter vergeben.

**ABBILDUNG 2:**
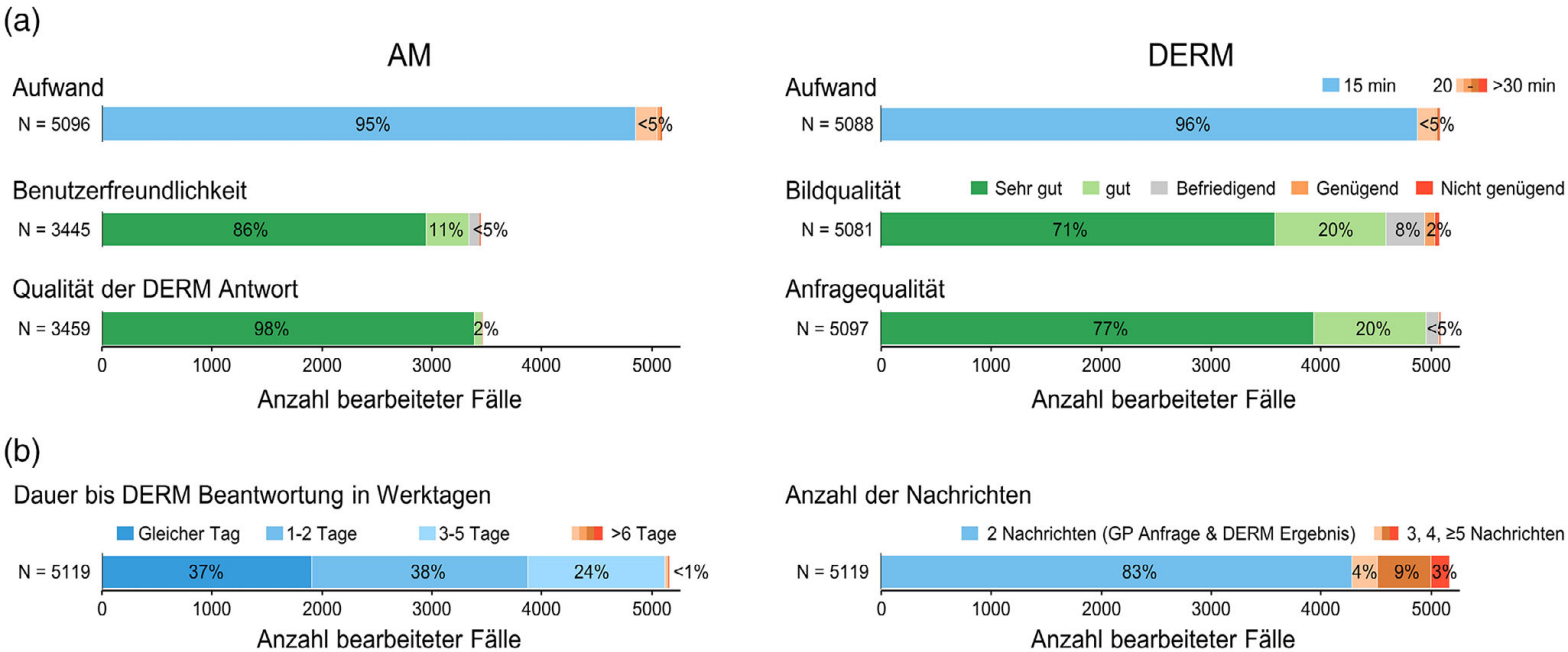
Qualität, Aufwand und Zufriedenheit der Allgemeinmediziner und Dermatologen mit dem teledermatologischen System. (a) Zusammenfassung der im System erfassten fallspezifischen Bewertungen in Prozent aller ausgefüllten Fälle. (b) Zusammenfassung der Bearbeitungsdauer durch Dermatologen in Arbeitstagen (abzüglich Wochenenden und Feiertagen) sowie der Anzahl der ausgetauschten Nachrichten pro Fall. Es wurde nicht zwischen medizinischen Rückfragen und Dankesnachrichten unterschieden.

### Bewertung des teledermatologischen Systems aus Sicht der Allgemeinmediziner und Dermatologen

Allgemeinmediziner und Dermatologen schlossen 95% respektive 96% der Fälle innerhalb von 15 Minuten ab (Abbildung 2). Die Allgemeinmediziner bewerteten die Benutzerfreundlichkeit mit 86 % als sehr gut (Benutzeroberfläche siehe Abbildung S1 im Online‐Supplement) und 98 % der Antworten der Dermatologen ebenfalls als sehr gut (Abbildung 2). Die Dermatologen beurteilten 71 % der Bilder und 77 % der Anfragen als sehr gut. Drei Viertel der Antworten der Dermatologen erfolgten binnen zwei Werktagen, und in 17 % der Fälle wurde die Chatfunktion für Rückfragen genutzt (Abbildung 2). Über die Zeit blieb die Nutzungshäufigkeit pro Allgemeinmediziner und Jahr stabil, ohne signifikante Veränderungen. Obwohl nur 38% der Allgemeinmediziner weiblich waren, übermittelten sie mit 2351 Fällen fast ebenso viele Fälle wie ihre männlichen Kollegen (2465 Fälle).

### Demographie, Diagnosegruppen und Triage

Die Geschlechterverteilung der 4361 Patienten zeigt, dass mit 56% mehr Patientinnen teilnahmen (Abbildung 3). Im Vergleich zur Gesamtbevölkerung Österreichs (Abbildung 3b) waren ältere Patienten überrepräsentiert, während jüngere unterrepräsentiert waren. Beide Bezirke zeigten nahezu deckungsgleiche demografische Verteilungen, mit Ausnahme der Kinder: Im Bezirk Liezen wurden relativ mehr Kinder eingeschlossen. Mit weiter bestehender Verfügbarkeit des teledermatologischen Systems stellten sich bis zu einem Drittel der Patienten erneut vor (Abbildung 3c), jedoch zu 69% mit einem anderen Hautproblem. Die gezeigten Beispielfälle repräsentieren Erkrankungen, die für Allgemeinmediziner im Allgemeinen ohne dermatologisches Fachwissen nicht lösbar sind (Abbildung 3d, e). Der pädiatrische Fall zeigt die Notwendigkeit der dermatoskopischen Bilder für die korrekte Diagnosestellung.

**ABBILDUNG 3:**
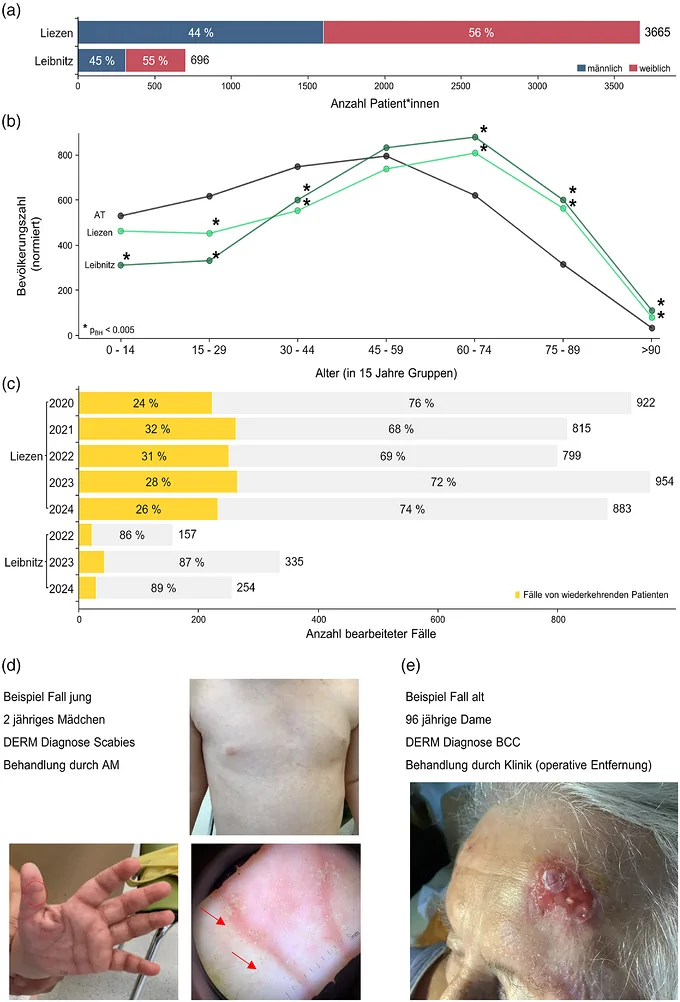
Übersicht über Patienten und Fälle. (a) Verteilung nach Geschlecht und Region. (b) Altersverteilung der Patienten im Vergleich zur österreichischen Gesamtbevölkerung (AT). Zur besseren Vergleichbarkeit wurden die Anzahlen von AT und Leibnitz jeweils auf die Gesamtanzahl in Liezen normiert. Der zweiseitige Chi‐Quadrat‐Test für Liezen vs. AT und Leibnitz vs. AT war signifikant (p < 0,001). Die Ergebnisse der *post hoc* paarweisen Vergleiche innerhalb der Altersgruppen mit 45–59 als Referenzgruppe und multipler Testkorrektur nach Benjamini‐Hochberg (BH) sind mit * gekennzeichnet (pBH < 0,005). (c) Verteilung der Fälle pro Region und Jahr, eingefärbt nach Anteil der wiederkehrenden Patienten. (d) Beispielfall einer Erkrankung, die nach teledermatologischer Diagnose direkt durch den Allgemeinmediziner behandelt werden kann. (e) Beispielfall einer Erkrankung, die nur an einer Klinik behandelt werden kann.

In 23% der 5119 Fälle stellten Allgemeinmediziner keine Verdachtsdiagnose, in 25% stimmte diese mit der Diagnose der Dermatologen überein und in 51% der Fälle wurde die Diagnose vom Dermatologen geändert (Abbildung 4a). Insgesamt waren in 19% keine Therapien nötig, während 61% von Allgemeinmedizinern behandelt wurden, 12% einen dermatologischen Normal‐, 3% einen Akut‐ und 2% einen Kliniktermin benötigten. Konsistent mit effizienter Triage konnten 80% der Fälle teledermatologisch gelöst werden, sodass nur 17% an Dermatologen oder eine Klinik weitergeleitet wurden (Abbildung 4b).

**ABBILDUNG 4:**
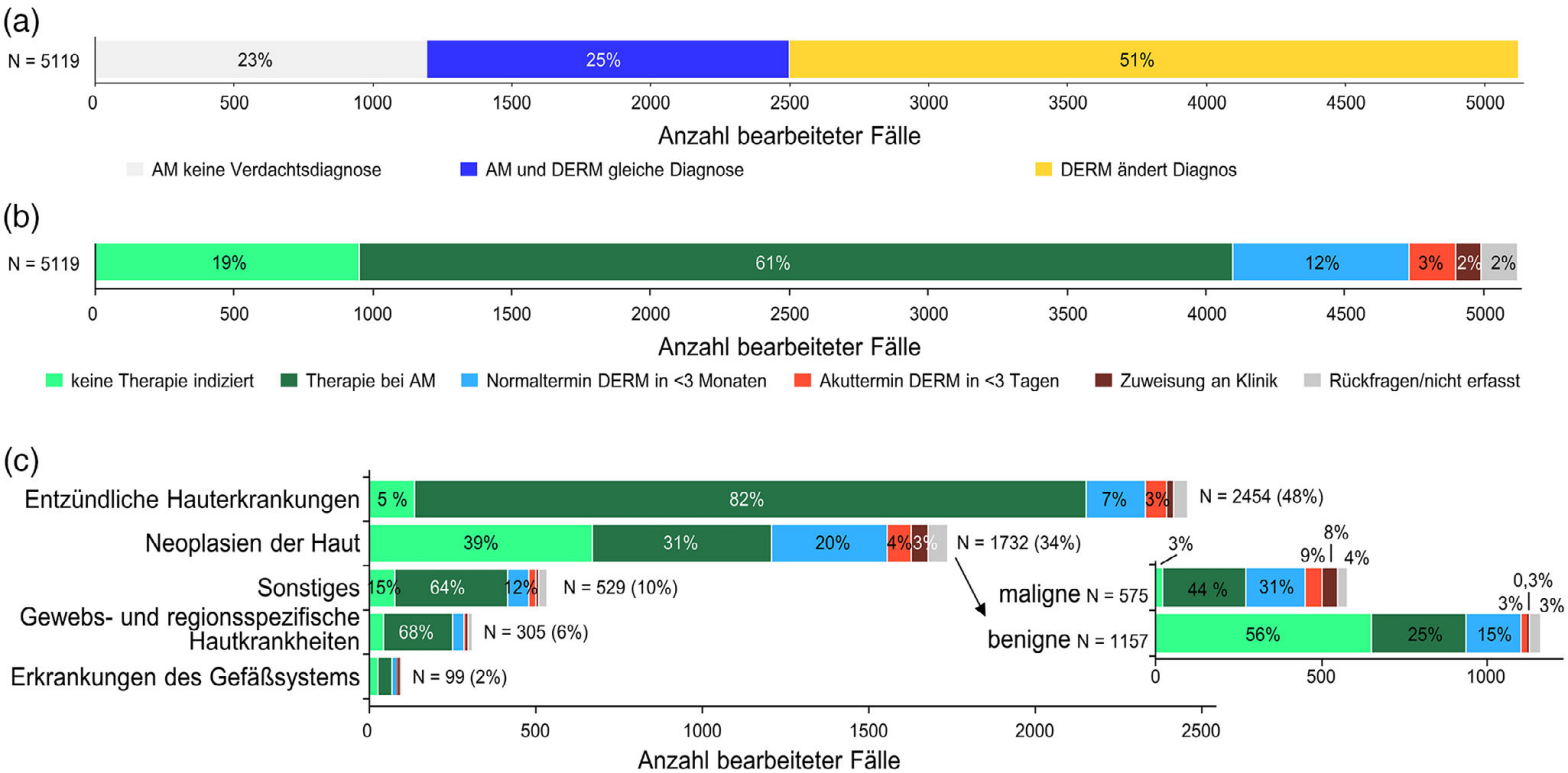
Teledermatologische Visiten ermöglichen eine effiziente Triage. (a) Vergleich der Verdachtsdiagnosen durch Allgemeinmediziner mit den Diagnosen der Dermatologen. (b) Übersicht über die weiterführende Behandlung aller Fälle entsprechend der Diagnose durch Dermatologen. (c) Verschiedene Diagnosegruppen unterscheiden sich deutlich hinsichtlich der erforderlichen weiteren Behandlungsschritte.

Die Kategorisierung der Gruppen zeigte, dass nur 34% (n = 1732) der Fälle Neoplasien betrafen, wovon 67% gutartig waren (n = 1157). Die malignen Neoplasien wurden von Allgemeinmedizinern (44%) oder Dermatologen (48%) behandelt. Nur 8 % der Patienten lehnten eine Behandlung ab, zum Beispiel aufgrund palliativer Versorgung. Weitaus mehr Anfragen (n = 3387) entfielen auf Erkrankungen im gesamten übrigen dermatovenerologischen Spektrum, die größtenteils eine Behandlung erforderten, jedoch ohne weitere Untersuchungen durch Dermatologen (Abbildung 4c).

Weder das Geschlecht der Patienten noch der Allgemeinmediziner hatte einen Einfluss auf die Häufigkeit der Therapie oder Zuweisungen (Abbildung S2 im Online‐Supplement). Die Behandlungsdringlichkeit unterschied sich beachtlich zwischen den Diagnosegruppen, wobei maligne Tumoren die höchste Dringlichkeit aufwiesen. Im Gegensatz dazu konnten entzündliche Hauterkrankungen inklusive entzündlicher Tumoren (zum Beispiel Dermatofibrome, Keloide, Viruswarzen, Abszesse) fast vollständig vom Allgemeinmediziner therapiert werden (Abbildung 5).

**ABBILDUNG 5:**
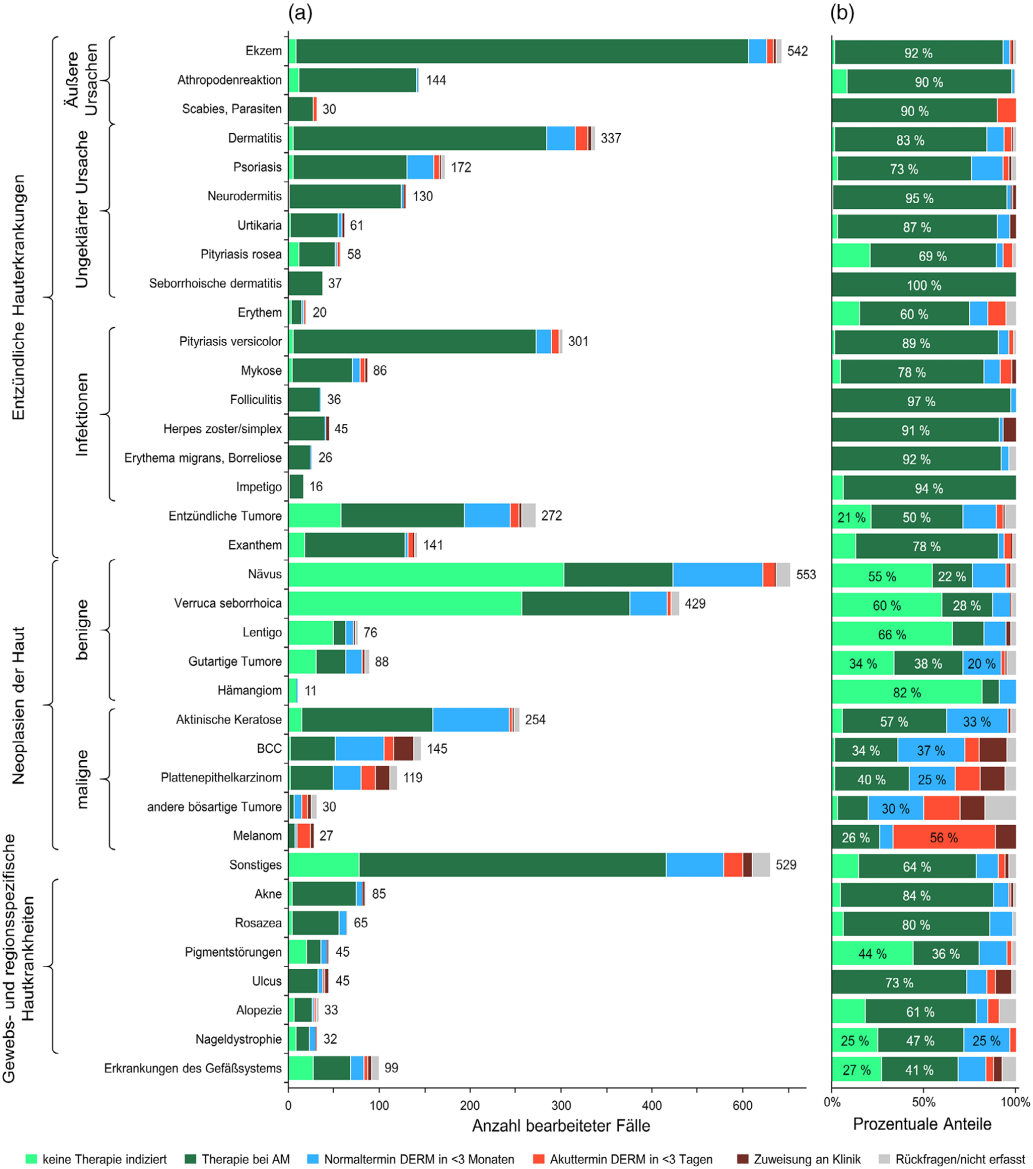
Unterschiedliche Dringlichkeit in Abhängigkeit von spezifischen Diagnosen. (a) Die Darstellung der Fallzahlen verdeutlicht die unterschiedliche Häufigkeit bestimmter Diagnosen. (b) Die prozentuale Darstellung der Weiterbehandlung innerhalb jeder Diagnose verdeutlicht die Unterschiede in der Dringlichkeit einer fachärztlichen Überweisung. Auffallend ist der hohe Prozentsatz an „Therapie beim Allgemeinmediziner“ bei entzündlichen Erkrankungen; fachärztliche Therapieempfehlungen wurden durch Allgemeinmediziner umgesetzt.

### Auswertung der Fragebögen

Von den 3665 ausgeteilten Fragebögen wurden 692 (18,8%) retourniert. Es gab Rückläufer aus allen Altersgruppen, Geschlechtern und Bildungsniveaus (Abbildung S3 im Online‐Supplement). Dabei antworteten mehr Patientinnen (48% weiblich, 38% männlich). Insgesamt waren 95% der Patienten mit der Teledermatologie zufrieden. Nach Behandlung waren 87% mit dem Ergebnis zufrieden und nur 1% unzufrieden, während 12% keine Angabe machten, teils, weil die Behandlung noch nicht begonnen hatte.

Das Freitextfeld „Größte Vorteile“ wurde von 77% ausgefüllt. Die meistgenannten Vorteile waren Verfügbarkeit, Bequemlichkeit, Vertrauen in den Allgemeinmediziner und die Behandlungsqualität/‐geschwindigkeit. Nachteile wurden in 96% keine genannt, während 3% den fehlenden persönlichen Kontakt zum Dermatologen kritisierten. Während die meisten Patienten privat (89%) oder öffentlich (3%) anreisten, benötigten 2,3% (14 Patienten) einen Ambulanzwagen oder einen Krankentransport (Abbildung S3 im Online‐Supplement). Insgesamt 34 Patienten teilten persönliche Kommentare mit, die Dank, Lob oder den Wunsch nach Ausbau enthielten.

Die durchschnittliche Zufriedenheit der Allgemeinmediziner lag bei 1,25 (sehr gut), wobei in den zurückgesandten Fragebögen (51%, n = 16) nur einmal 3 (befriedigend) vergeben wurde. Alle Allgemeinmediziner nannten Vorteile, wobei vor allem Schnelligkeit, Zeitersparnis, rasche Abwicklung, niederschwellige Diagnosesicherung und Behandlungsempfehlung sowie ein Lerneffekt hervorgehoben wurden. Sieben Allgemeinmediziner nutzten die Chat‐Funktion und waren mit den Antworten vollkommen zufrieden. Fast alle Allgemeinmediziner (15 von 16) sprachen sich eindeutig für das teledermatologische System aus und würden es Kollegen weiterempfehlen.

Unter den 23% retournierten Fragebögen (n = 20 von 86) von Allgemeinmedizinern ohne Teledermatologie hatten lediglich vier keine Kenntnis vom Projekt, während 15 es gerne nutzen würden. Allgemeinmediziner ohne Teledermatologie erwarteten ähnliche Vorteile, wie sie von den teilnehmenden Allgemeinmedizinern genannt wurden, und betonten zusätzlich die Krankenhausanbindung. Allerdings wurden von ihnen mehr Nachteile genannt: fehlender Patientenkontakt, Limitierung der Diagnosestellung durch unzureichende Bildqualität und erhöhter administrativer Aufwand.

## DISKUSSION

Unser Ziel war es, eine pragmatische Lösung für die ländlichen Versorgungsengpässe zu entwickeln, die durch die hohe Prävalenz dermatologischer Erkrankungen^24^, ^25^, ^26^, ^27^ und die steigende dermatologische Versorgungslast bedingt sind.^20^ Das aktuelle Überweisungssystem vom Allgemeinmediziner zum Dermatologen verursacht lange Wartezeiten, was bei kritischen Erkrankungen mit hohem Leid und Kosten verbunden ist.

Insgesamt wurden 35 Allgemeinmediziner über eine SF‐Technologie digital mit vier Dermatologen vernetzt; die Kommunikation erfolgte ausschließlich zwischen Ärzten. Ziel war es, die interdisziplinäre Zusammenarbeit zu verbessern und mittels einer am Schweregrad orientierten Triage einen beschleunigten Zugang zur dermatologischen Versorgung zu sichern. Diese Triage, vergleichbar mit den Vorgehensweisen in Notaufnahmen, priorisiert Dringlichkeit und Versorgungsart, hat jedoch keinen Einfluss auf die Gesamtanzahl der Überweisungen. Die SF‐Teledermatologie wurde entwickelt, um bestehende Netzwerke zu stärken, Patientenströme unverändert zu erhalten, Zeit und Wege zu sparen und die dermatologische Versorgung zu verbessern.

Daher gab es keinen persönlichen Kontakt zwischen Patienten und Dermatologen. Stattdessen wurde ihr Fall, einschließlich Bildern und Anamnese, zur Beurteilung an die Dermatologen gesendet, sofern dies vom Allgemeinmediziner als notwendig erachtet wurde. Die bisherige Überweisung zum Dermatologen wurde so durch eine digitale ersetzt. Nach Beantwortung des Falles teilten die Allgemeinmediziner die Diagnose mit und übernahmen die Behandlung. Dies stellte eine zeitnahe Therapie und Betreuung der Betroffenen sicher, sodass sie mit ihrer Erkrankung nicht alleingelassen wurden. Gerade die fehlende Betreuung ist ein großes Problem bei internetbasierten Selbstdiagnosen oder medizinischen Beratungen, bei denen sowohl vertrauenswürdige als auch fragwürdige Dienste koexistieren. Selbst diagnostisch als sehr zuverlässig geltende künstliche Intelligenz (KI)‐unterstützte Tools tragen oftmals weniger zur Beruhigung als zur Verunsicherung der Betroffenen bei.^19^, ^28^ Studien zeigten, dass Patienten 40%–80% des Besprochenen kurz nach Verlassen der Praxis vergessen haben.^29^ Dies unterstreicht die Schlüsselrolle der Allgemeinmediziner, welche die primäre Versorgung sicherstellen, relevante Informationen dokumentieren und Details bei Bedarf erneut erläutern.

Dieses Projekt etablierte erfolgreich einen teledermatologischen Service im niedergelassenen Bereich, der bestehende Strukturen nutzt und bereichert. Einzigartig ist die Zusammenarbeit zwischen Politik, Gesundheitskassen, Ärztevertretung, Ärzten aus dem niedergelassenen Bereich wie auch der Klinik. Die fünfjährige Laufzeit mit fallbasierter Einzelauflösung in 5119 Fällen und 692 retournierten Patientenfragebögen ist besonders hervorzuheben. Diese Studie zeichnet sich gegenüber anderen großen Studien durch detaillierte Falldaten aus, wie der kategorisierten Vorgehensweise von „keiner Therapie“ bis zur „dringenden Überweisung“ und der Bewertung von Zeitaufwand und Qualität.^30^, ^31^, ^32^

Die Diskrepanz zwischen den Verdachtsdiagnosen der Allgemeinmediziner und der Beurteilung durch Dermatologen war deutlich ausgeprägt. Nur 25% der Fälle wurden vom Dermatologen bestätigt, 51% wurden korrigiert und in 23% wurde keine Verdachtsdiagnose vom Allgemeinmediziner geäußert. Ähnlich zeigte sich in der pädiatrischen Teledermatologie, dass 32 % aller Fälle von den Dermatologen bestätigt wurden, wobei 60 % der 53,7 % der Fälle mit Verdachtsdiagnose durch Kinderärzte bestätigt wurden.^33^ Diese diagnostische Lücke verdeutlicht die Herausforderungen der Allgemeinmediziner in der Betreuung dermatologischer Erkrankungen und betont die kritische Funktion der Dermatologen bei der gemeinsamen Patiententriage, der korrekten Diagnose und Behandlung. Aus Patientensicht profitierten 80% dank Teledermatologie von einer schnelleren Diagnose und Behandlung und sparten sich oft monatelange Wartezeiten und längere Anfahrtswege im Vergleich zum Standardweg. Aus dieser deskriptiven Studie ist es nicht möglich zu beurteilen, ob diese Form der Teledermatologie die Anzahl an Überweisungen senkt. Das vor diesem Projekt bestehende dermatologische Versorgungsdefizit könnte die Überweisungen gesteigert haben, da zuvor ignorierte oder tolerierte Erkrankungen nun behandelt wurden.

In 61% der Fälle wurde die Therapie durch Allgemeinmediziner entsprechend den Empfehlungen der Dermatologen durchgeführt, am häufigsten bei entzündlichen Hauterkrankungen. In 19% der Fälle war keine Therapie indiziert, meistens bei gutartigen Tumoren. Dagegen benötigten 17% der Fälle eine persönliche Vorstellung. Diese Verteilungsverhältnisse waren über die gesamte Laufzeit stabil.

Die Auswertung der Diagnosegruppen zeigte einen unerwartet geringen Anteil von Tumoren (ein Drittel), wovon wiederum nur ein Drittel maligne waren. Die aktinische Keratose war der häufigste maligne Tumor und wurde weitgehend von Allgemeinmedizinern behandelt. Die größte Diagnosegruppe waren Dermatosen, überwiegend Entzündungen, und erforderten größtenteils eine Therapie, die primär von Allgemeinmedizinern durchgeführt wurde.

Einige Dermatosen verursachen am Anfang wenig Leidensdruck, was oft zu einer späten Diagnose und daraus resultierenden Folgeschäden führt. Dazu zählen bösartige Tumoren im Frühstadium, aber auch infektiöse Erkrankungen wie die Borreliose, die in 26 Fällen diagnostiziert wurden und mittels Antibiotikatherapie heilbar sind. Unbehandelt schädigen Borreliosen jedoch diverse Organsysteme, was Invalidität zur Folge hat und in Österreich die Anerkennung als Berufsdermatose begründet. Ebenso reduzieren rechtzeitige Diagnosen und Behandlungen bei Tumoren, die durch chronische UV‐Exposition verursacht werden und seit diesem Jahr als Berufsdermatosen anerkannt sind, die Gesundheits‐ und öffentlichen Kosten erheblich. Einsparungspotenzial findet sich im Patiententransport, da 2,3% auf Krankentransporte oder Krankenwagen angewiesen sind (Abbildung S3 im Online‐Supplement).

In Leibnitz wurden auffallend weniger Fälle eingereicht als in Liezen, was auf die bessere dermatologische Versorgung durch die räumliche Nähe zu Graz hinweist. Dies impliziert, dass die Teledermatologie nur bei tatsächlichem Bedarf, vor allem in unterversorgten, dünn besiedelten Regionen, genutzt wurde.

Als eine Limitation in der Telemedizin wurde in früheren Publikationen die „digitale Ungleichheit“ sozialer und geographischer Natur beschrieben, die nur einer bestimmten Klientel den Zugang ermöglicht. ^34^ Diese Ungleichheit wird durch die direkte digitale Anbindung von Allgemeinmedizinern und Dermatologen ausgeglichen. Auch die der SF‐Teledermatologie inhärenten Nachteile^35^ bezüglich Anamneseerhebung, persönlicher Untersuchung und direkter Befundbesprechung konnten durch die Einbindung der kundigen Allgemeinmediziner alltagstauglich gelöst werden. Mit der teledermatologischen Anbindung an die Universitätsklinik für Dermatologie in Graz konnte erreicht werden, dass in jedem Fall der nächste Behandlungsschritt eingeleitet werden konnte. Im Zweifelsfall beruhten Diagnose und Therapieempfehlung auf einem breiteren ärztlichen Konsens. Keiner der Beteiligten – weder Patienten noch Allgemeinmediziner oder Dermatologen – wurde mit einem Problem alleingelassen. Im Gegensatz dazu konnten in der Studie von Otten aus dem Jahr 2023 zu einem deutschen teledermatologischen Service 70 % der Fälle teledermatologisch geklärt werden, und nur 53 % wurden zur Zufriedenheit gelöst.^36^

In unserem Projekt bewerteten 95% der Patienten den teledermatologischen Service positiv und 87% waren mit der Behandlung ihrer Hautprobleme zufrieden. Mit einer Rücklaufquote von nur 18,8% liegt die Studie im Bereich der typischen Antwortraten.^36^ Dennoch kann die hohe Anzahl von 692 Antworten als repräsentative Stichprobe betrachtet werden. Nichtsdestotrotz kann eine Verzerrung der Ergebnisse durch fehlende Rückmeldungen – vor allem von Patienten mit negativen oder neutralen Erfahrungen sowie von Patienten ohne klare Bewertung ihrer Behandlungserfahrung – nicht ausgeschlossen werden.

Die Akzeptanz und Zufriedenheit mit der Teledermatologie dürfte maßgeblich durch die COVID‐19‐Pandemie beeinflusst worden sein.^13^ Die Notwendigkeit, während der *Lockdowns* physischen Kontakt zu vermeiden, erhöhte die Zahl telemedizinischer Visiten^37^ sowie deren Akzeptanz und die Digitalisierung. Gleichzeitig limitierte der starke Rückgang der persönlichen Hausarztbesuche^37^ die potenziellen Teledermatologie‐Fälle. Der Gesamteffekt lässt sich hier jedoch nur schwer beurteilen, da präpandemische Daten fehlen und während der Pandemiejahre kontinuierlich weitere Allgemeinmediziner eingeschlossen wurden.

Für Dermatologen ist die Teledermatologie bei entsprechender Planung gut in den fachärztlichen Arbeitsalltag integrierbar. Im Gegensatz zum direkten Patientenkontakt im oft hektischen Ordinations‐Setting erlaubt die SF‐Teledermatologie ein fokussiertes und konzentriertes Arbeiten ohne Unterbrechungen. Während der administrative Aufwand für Dermatologen im Vergleich zur persönlichen Visite leicht sinkt, steigt die Arbeitsbelastung für Allgemeinmediziner. Auffallend ist, dass die gesteigerte Arbeitslast von den aktiv teilnehmenden Allgemeinmedizinern seltener als Nachteil wahrgenommen wurde als nicht teilnehmende Allgemeinmedizinern. Die Effizienzgewinne durch den Triage‐Effekt – mit deutlich früherer Diagnose und Intervention durch Dermatologen – gleichen die zusätzliche Arbeitsbelastung wieder aus, und das nicht nur aus gesellschaftlicher, sondern offenbar auch aus Sicht der Allgemeinmediziner. Dementsprechend bewerteten über 90 % der Allgemeinmediziner und Dermatologen die Benutzerfreundlichkeit als sehr gut oder gut. Die Effizienz war bemerkenswert, denn über 95 % der Fälle wurden innerhalb von 15 Minuten eingereicht (Allgemeinmediziner) oder beantwortet (Dermatologen).

Während die Schulung der Allgemeinmediziner als potenzielle Einschränkung gesehen werden könnte, zeigte unsere Studie nur wenige Bilder von unzureichender Qualität, obwohl die Allgemeinmediziner lediglich eine minimale, einmalige Schulung in Dermatoskopie erhielten und jederzeit Zugriff auf ein 1:27‐minütiges Schulungsvideo hatten. Dies bekräftigt die Praxistauglichkeit der dermatoskopiegestützten Teledermatologie unter realen Bedingungen. Der Erfolg ist wahrscheinlich darauf zurückzuführen, dass ausschließlich medizinische Fachkräfte und keine Patienten in den Prozess involviert waren. Bei mangelhafter Bildqualität lag es in der Verantwortung des Dermatologen, bessere Bilder nachzufordern, spezifische Anleitungen zur Verbesserung zu geben oder die Patienten direkt zur persönlichen Visite zu bestellen.

Die Verantwortung für die korrekte Umsetzung der dermatologischen Empfehlungen liegt hingegen beim Allgemeinmediziner. Beiden Parteien wird daher zu einer entsprechenden Erweiterung der Haftpflichtversicherung geraten. In fünf Jahren wurde allerdings kein einziger Haftungsfall verzeichnet.

Diese durchgehend positiven Erfahrungen haben die Autoren, Allgemeinmediziner und Dermatologen während des gesamten Projekts stark motiviert und erklären zugleich dessen Erfolg: Eine Versorgungslücke wurde geschlossen, regionale Strukturen wurden genutzt und Kosten erstattet. Nicht zuletzt basiert der Erfolg auch auf der guten und konstruktiven Planung und dem Willen zum Gelingen des Projektes seitens aller Beteiligter. Zukünftig könnte die Ausweitung des teledermatologischen Dienstes auf andere Primärversorger wie Kinderärzte^33^ die geschaffene Infrastruktur nutzen, um die Versorgung stark betroffener Patientengruppen zu verbessern. Ebenso würde die Einbindung von Urologen und Gynäkologen die Versorgung von Patienten mit stark betroffenen, schambehafteten Körperregionen verbessern. Gleichzeitig könnte die Einbindung von Gesundheitspersonal in Justizvollzugsanstalten Zugangshürden abbauen.^38^

Die Vergütung der ärztlichen Leistung durch die Krankenkassen ist einmalig, entspricht aber nicht dem tatsächlichen Arbeitsaufwand. Sie liegt etwas unter dem Wert, der in einer vergleichbaren Studie aus Australien 2018 veröffentlicht wurde.^39^ Es muss aber festgehalten werden, dass in dieser Situation lediglich Tumoren begutachtet wurden. Die Behandlung komplexerer Dermatosen durch Allgemeinmediziner, wie sie in unserem Projekt praktiziert wurde, fand nicht statt. Umso deutlicher wird daher der Wert dieses Projekts: Ein teledermatologisches Werkzeug mit gesetzeskonformer Sicherheit unterstützt Allgemeinmediziner und Dermatologen auf einfache Weise dabei, alle Fälle zu bearbeiten und einer Lösung zuzuführen. Eine direkte Arzt‐zu‐Arzt‐Kommunikation auf Augenhöhe löst reale Probleme und ist mittlerweile im Alltag angekommen.

### Limitationen

Diese deskriptive Studie mit einem nichtrandomisierten Design ohne Referenzregion oder Kontrollgruppe kann die teledermatologische Intervention nur begrenzt direkt mit der Standardversorgung vergleichen oder den Einfluss des Projekts quantifizieren. Kontrollgruppen wurden aufgrund ethischer Bedenken hinsichtlich des Vorenthaltens von Versorgung nicht eingesetzt. Referenzregionen waren aufgrund der logistischen Komplexität sowie der großen regionalen Unterschiede in Österreich beim Zugang zur Gesundheitsversorgung und der Topographie, die das Patientenverhalten beeinflussen, nicht realisierbar. Dementsprechend konnten Daten zur tatsächlichen dermatologischen Zuweisungsrate nicht erhoben werden. Darüber hinaus erfolgte keine unabhängige Überprüfung der Diagnosen und die Behandlungserfolge wurden nicht über die subjektiven Rückmeldungen der Patienten hinausgehend weiterverfolgt. Dies begrenzt die Aussagekraft bezüglich der Diagnosegenauigkeit und der Wirksamkeit der Therapien. Der Beobachtungszeitraum umfasst sowohl die Zeit der Coronapandemie als auch die Jahre danach, wobei der Einfluss der Pandemie auf die Fallzahlen aufgrund des kontinuierlichen Aufbaus nicht klar herausgefiltert werden konnte.

Der Rücklauf der Patientenfragebögen war begrenzt und könnte daher einer Verzerrung durch Nichtantwort unterliegen. Dennoch sollte die hohe Anzahl an Antworten eine repräsentative Stichprobe gewährleisten. Das dichotome Format (ja/nein) der Zufriedenheitsfragen war effektiv für einen generellen Überblick, erlaubte jedoch keine nuancierte Erfassung der Zufriedenheit. Die Verwendung nicht validierter Fragebögen ermöglichte eine flexible Anpassung der Terminologie an die Patientenkohorte und war unvermeidbar, da zum Projektstart keine für Telemedizin bzw. Teledermatologie spezifischen, validierten deutschen Fragebögen zur Verfügung standen.

## DANKSAGUNG

Wir danken herzlich allen Patienten, allen Allgemeinmedizinern (Heidelinde Altenaichinger, Anneliese Auer, Wolfgang Auer, Dominik Augustin, Philipp Brodatsch, Claudia Burgstaller, Astrid Doppler, Jakob Dorner, Pia Edlinger, Walter Gsöllpointner, Daniela Habersatter‐Theil, Markus Hamp, Martin Hasibeter, Johann Holler, Norbert Holzmüller, Renate Jocham, Thomas Kappaun, Klaus Karrer, Oliver Lammel, Manfred Manninger, Doris Nestl‐Treiber, Magdalena Putz, Martina Rauscher, Richard Rezar, Josef Rampler, Alexander Schwarz, Peter Sigmund, Christian Soral, Robert Sponner, Alexander Stradner, Maria Thier, Sabine Trummer‐Grün, Renate Wiesler, Markus Wippel, Thomas Zorn, Gudrun Zweiker) und allen Dermatologen (Urban Cerpes, Peter Kahofer, Thomas Kainz, Manfred Tritscher) für die jahrelange motivierte Unterstützung, das wertvolle Feedback und die Datenspende. Wir danken herzlich Dietmar Bayer für die initiale und fortwährende wertvolle Unterstützung, Franz Schuller für den hervorragenden technischen und organisatorischen Support und Horst Stuhlpfarrer für die rechtliche Begleitung und guten Ideen im Projektdesigns. Wir sind den Reviewern für ihre wertvollen und konstruktiven Rückmeldungen und Lisa Otto für die professionelle sprachliche Überarbeitung äußerst dankbar.

## INTERESSENKONFLIKT

R.H.‐W. ist Miteigentümer der Teledermatologie‐Firma e‐derm‐consult, Graz, Österreich. Die übrigen Autoren erklären keinen Interessenkonflikt.

Open access funding provided by Medizinische Universitat Graz/KEMÖ.

## ERKLÄRUNG ZU GENERATIVER KI UND KI‐GESTÜTZTEN TECHNOLOGIEN

Bei der Erstellung dieses Manuskripts haben die Autoren *Large Language Models* (LLM, zum Beispiel Gemini, DeepL, GPT‐4o) zur Unterstützung der Übersetzung sowie zur Verbesserung der Lesbarkeit einzelner Textteile verwendet. Die Autoren überprüften und redigierten den Text anschließend sorgfältig und übernehmen die volle Verantwortung für den Inhalt der Veröffentlichung.

## Supporting information



Supplementary information

Supplementary information
