# Supplementary material for: Effiziente Triage und verbesserte Patientenversorgung: Fünf Jahre Teledermatologie in der Steiermark: Efficient triage and improved patient care: Five years of teledermatology in Styria
Source: J Dtsch Dermatol Ges. 2026 Nov 10;24(11):1598–609. [Article in German] doi: 10.1111/ddg.70097x (PMC13749600; doi:10.1111/ddg.70097x)
Supplement: Supplementary file 1 [file DDG-24-1598-s001.docx]

Online Appendix 1

Effiziente Triage und verbesserte Patient*innenversorgung: Fünf Jahre Teledermatologie in der Steiermark

Arzberger Edith^1,*,#^, Bordag Natalie^1,#^, Hofmann-Wellenhof Elena^1^, Freytag Leo^1^, Hofmann-Wellenhof Rainer^1,2^

^1^Univ.-Klinik für Dermatologie und Venerologie, Medizinische Universität Graz, Graz, Österreich

^2^e-derm-consult, Graz, Österreich

^#^geteilte Erstautorenschaft

*Korrespondenzadresse: PD Dr. Edith Arzberger; [edith.arzberger@medunigraz.at](mailto:edith.arzberger@medunigraz.at); Auenbruggerplatz 8, 8036 Graz

JDDG: Journal der Deutschen Dermatologischen Gesellschaft. 2026;1-10

<https://doi.org/10.1111/ddg.70097>

**Inhaltsverzeichnis**

[Sicherheit	2](#_Toc212491423)

[Online Abbildung S1	3](#_Toc212491424)

[Online Abbildung S2	4](#_Toc212491425)

[Online Abbildung S3	5](#_Toc212491426)

[Fragebogen Patient*innen nach abgeschlossener Behandlung	6](#_Toc212491427)

[Fragebogen AM mit Teledermatologie	8](#_Toc212491428)

[Fragebogen AM ohne Teledermatologie	11](#_Toc212491429)

# Sicherheit

Die von E-derm-consult bereitgestellte Anwendung ist Datenschutz-Grundverordnung (DSGVO) konform, es erfolgen jährlich externe Audits und das Unternehmen ist ISO 9001-2008 und ISO 13485:2003 zertifiziert. Sowohl AM als auch DERM können ausschließlich vom technischen Administrator registriert werden und loggen sich stets per Benutzername/Passwort von jedem internetfähigen Gerät mittels Zwei-Stufen-Authentifizierung an.

Die Datenübertragung erfolgt verschlüsselt (HTTPS-Verbindung) und sämtliche patientenbezogenen Daten und Bilder werden ausschließlich auf den Servern der Firma E-derm-consult gespeichert, nicht am iPad. Die Datenbestände werden täglich, wöchentlich, monatlich und jährlich revolvierend auf einem separatem Server gesichert. Die Datenbanken sind grundsätzlich auf der Ebene der Datendatei verschlüsselt, Exports für wissenschaftliche Analysen können nur über einen Schlüssel freigegeben werden. Passwörter werden grundsätzlich als Hash gespeichert.

# Online Abbildung S1

**Online Abbildung S1: Einblicke in die graphische Nutzeroberfläche aus Sicht der AM (3, 4, 6) und aus Sicht der DERM (3, 5).** Die Nummern verweisen auf die entsprechenden Schritte wie auch in Abb. 1 gezeigt.

# Online Abbildung S2

**Online Abbildung S2: Die weitere Verfahrensweise ist unabhängig vom Geschlecht der AM oder Patient*innen. (A)** Die weitere Verfahrensweise ist unabhängig vom Geschlecht der Patient*innen. **(B)** Die weitere Verfahrensweise ist auch unabhängig vom Geschlecht der AM.

# Online Abbildung S3

**Online Abbildung S3: Patient*innen Fragebögen Ergebnisse.** **(A)** Demographische Verteilung der Patient*innen. **(B)** Ergebnisse zum Nutzungsverhalten und Zufriedenheit der Patient*innen aus den verwertbaren Rückläufern. kA – keine Angabe.

# Fragebogen Patient*innen nach abgeschlossener Behandlung

**„Teledermatologie-Projekt Steiermark“**

**Fragebogen für Patienten und Patientinnen**

1. **Haben Sie aufgrund eines Hautproblems Ihren praktischen Arzt/Ihre praktische Ärztin aufgesucht?**

O	ja	O	nein	(bitte Zutreffendes ankreuzen)

**WENN JA: Aus welchem Grund haben Sie nicht von vornherein einen Hautarzt/eine Hautärztin oder eine dermatologische Ambulanz aufgesucht?**

…………………………………………………………………………………………………………………………………………..

2. **Wussten Sie im Vorfeld davon, dass Ihr Arzt/Ihre Ärztin im „Teledermatologie-Projekt Steiermark“ mitwirkt?**

O ja	O nein	(bitte Zutreffendes ankreuzen)

**WENN JA: Wie wurden Sie darauf aufmerksam?...............................................................**

3. **Waren Sie prinzipiell mit dem teledermatologischen Service zufrieden?**

O…..ja	O…..nein	(bitte Zutreffendes ankreuzen)

4. **Worin sehen Sie für sich den größten VORTEIL im „Teledermatologie-Projekt Steiermark“?**

…………………………………………………………………………………………………………………………………………..

**Worin sehen Sie für sich den größten NACHTEIL im „Teledermatologie-Projekt Steiermark“?**

…………………………………………………………………………………………………………………………………………..

**Wurden Ihre Hautprobleme zu Ihrer Zufriedenheit abgeklärt und behandelt?**

O	ja	O	nein	(bitte Zutreffendes ankreuzen)

**WENN NEIN: Aus welchem Grund?** ......................................................................................

…………………………………………………………………………………………………………………………………………..

5. **Hat Ihr Hausarzt/Ihre Hausärztin Sie persönlich oder telefonisch über den fachärztlichen Befund informiert?**

O	persönlich	O	telefonisch	O	andere Person	O	gar nicht

(bitte Zutreffendes ankreuzen)	Bitte das Blatt wenden!

6. **Fahren Sie (üblicherweise) privat, mit öffentlichen Verkehrsmitteln oder mit Krankentaxi/Rettung zu Ihrem Arzt/Ihrer Ärztin?**

O	privat	O	öffentlich	O	Krankentaxi/Rettung	(bitte ankreuzen)

7. **Wie weit sind die Ordination des Hausarztes/der Hausärztin bzw. des Dermatologen/der Dermatologin von Ihrem Zuhause entfernt?**

……………..Kilometer (Hausarzt/- ärztin)	………………………Kilometer (Facharzt, -ärztin)

(bitte ungefähre Kilometerangabe einfügen)

8. **Denken Sie, dass Sie durch das „Teledermatologie-Projekt Steiermark“ schneller zu einem Befund bzw. einer notwendigen Behandlung gekommen sind?**

O…..ja	O…..nein	(bitte Zutreffendes ankreuzen)

**WENN JA: Wie hoch schätzen Sie Ihre Zeitersparnis ein?**

Zeit bis zu einem Termin beim Facharzt/bei der Fachärztin…………………………………………….

Zeit im Wartezimmer beim Facharzt/bei der Fachärztin……………………………………..…………..

**Was ich noch sagen wollte:**

**…………………………………………………………………………………………………………………………………………..**

**…………………………………………………………………………………………………………………………………………..**

Meine Teilnahme am „Teledermatologie-Projekt Steiermark“ war…………………………………….(Monat/Jahr)

Weitere Angaben zur Person:	Alter………………	O…..weiblich	O	männlich

Ausbildung:	O	Pflichtschule	O…..(berufsbildende) höhere Schule	O	FH/Hochschule/Universität

Herzlichen Dank für Ihre Zeit!

**Bitte geben Sie den ausgefüllten Fragebogen bei Ihrem Hausarzt/Ihrer Hausärztin ab!**

# Fragebogen AM mit Teledermatologie

**Fragebogen Hausarzt/Hausärztin**

In der folgenden Umfrage geht es um das Thema „Teledermatologie aus der Sicht des Hausarztes/der Hausärztin und des Patienten/der Patientin

**Name:**	(*Angabe freiwillig*)

**Datum:**

**Frage 1:**

| Waren Sie mit dem teledermatologischen Service zufrieden? | | | | |
| --- | --- | --- | --- | --- |
|  |  |  |  |  |
| ☐ Stimme zu | ☐ Stimme eher zu | ☐ Teils, teils | ☐ Stimme eher nicht zu | ☐ Stimme nicht zu |

**Frage 2:**

| Wenn es Probleme gab, sind diese Probleme im Bereich der medizinischen Anfrage oder im Bereich der technischen Abwicklung begründet? | |
| --- | --- |
|  |  |
| ☐ technische Abwicklung | ☐ medizinische Anfrage |

**Frage 3:**

Welche Vorteile sehen Sie in diesem Projekt?

*(Platz für eine Freitext Antwort)*

**Frage 4:**

Welche Nachteile sehen Sie in diesem Projekt?

*(Platz für eine Freitext Antwort)*

**Frage 5:**

| Waren Sie mit der Schnelligkeit der Bearbeitung Ihrer Anfrage zufrieden? | | | | |
| --- | --- | --- | --- | --- |
|  |  |  |  |  |
| ☐ Stimme zu | ☐ Stimme eher zu | ☐ Teils, teils | ☐ Stimme eher nicht zu | ☐ Stimme nicht zu |

**Frage 6:**

| Haben Sie Rückfragen zur Diagnose und/oder zur Beantwortung Ihrer Anfrage gestellt? | |
| --- | --- |
|  |  |
| ☐ Ja | ☐ Nein |

Wenn ja, bei wie vielen Fällen zirka? (Angabe in Prozent).......................%

**Frage 7:**

| Wenn ja, waren Sie mit der Beantwortung Ihrer Rückfrage zufrieden? | | | | |
| --- | --- | --- | --- | --- |
|  |  |  |  |  |
| ☐ Stimme zu | ☐ Stimme eher zu | ☐ Teils, teils | ☐ Stimme eher nicht zu | ☐ Stimme nicht zu |

**Frage 8:**

| Würden Sie das Teledermatologie-Projekt wieder in Anspruch nehmen? | |
| --- | --- |
|  |  |
| ☐ Ja | ☐ Nein |

**Frage 9:**

| Würden Sie das Teledermatologie-Projekt Ihren Kollegen/Kolleginnen weiterempfehlen? | |
| --- | --- |
|  |  |
| ☐ Ja | ☐ Nein |

**Frage 10:**

| Wie war der Ablauf bei der Behandlung der Patienten/Patientinnen mit dermatologischen Problemen? | |
| --- | --- |
|  |  |
|  | *Rangordnung (1- 4):* |
| Patienten/Patientinnen selbst behandelt (ohne Tele) |  |
| Patienten/Patientinnen zum Facharzt überwiesen |  |
| Teledermatologieservice sofort in Anspruch genommen |  |
| Patient/Patientin vorbehandelt und dann erst Teledermatologieservice in Anspruch genommen |  |

Hinweis: Ordnen Sie die Antworten in absteigender Wichtigkeit, indem Sie ihnen eine Zahl von 1-4 zuteilen.
1 ist hierbei die wichtigste und 4 die unwichtigste.

**Frage 11:**

Würden Sie die Möglichkeit einer Videokonsultation mit der Dermatologin/ dem Dermatologen nutzen?

| ☐ Ja | ☐ Nein |
| --- | --- |

**Frage 12:**

Würden sie das teledermatologische Service auch ohne Bezahlung anbieten?

| ☐ Ja | ☐ Nein |
| --- | --- |

Wenn nein: Welche Bezahlung pro Fall finden Sie angemessen?...........................................................

Vielen Dank für Ihre Teilnahme!

# Fragebogen AM ohne Teledermatologie

**Fragebogen Hausarzt/Hausärztin zum Projekt ‚Teledermatologie in der Steiermark‘**

**Name:**	(*Angabe freiwillig*)

**Datum:**

**Frage 1:**

| Kennen Sie das Projekt „Teledermatologie-Liezen“? | | | | |
| --- | --- | --- | --- | --- |
|  |  |  |  |  |
| ◻ Ja | ◻ Nur dem Namen nach | ◻ Nein |  |  |

**Frage 2:**

| Falls Nein, hätten Sie Interesse mehr darüber zu erfahren? | |
| --- | --- |
|  |  |
| ◻ Ja | ◻ Nein |

**Frage 3:**

| Falls Ja, haben Sie den Eindruck, dass der Tele-Service im Rahmen dieses Projektes zuverlässig ist? | |
| --- | --- |
|  |  |
| ◻ Ja | ◻ Nein |

**Frage 4:**

| Falls Ja, hätten Sie Interesse daran teilzunehmen? | |
| --- | --- |
|  |  |
| ◻ Ja | ◻ Nein |

**Frage 5:**

| Falls Ja, würden Sie das teledermatologische Service auch ohne Bezahlung anbieten? | |
| --- | --- |
|  |  |
| ◻ Ja | ◻ Nein |

**Frage 6**

Wenn nein: Welche Bezahlung finden Sie angemessen?

*(Platz für eine Freitext Antwort)*

**Frage 7:**

Welche Vorteile sehen Sie in diesem Projekt (für Sich/ und/oder die Patient*innen)

*(Platz für eine Freitext Antwort)*

**Frage 8:**

Welche Nachteile sehen Sie in diesem Projekt? (für Sich/ und/oder die Patient*innen)

*(Platz für eine Freitext Antwort)*

Vielen Dank für Ihre Teilnahme!
